# Dietary Acid Load (DAL), Glycated Hemoglobin A1c (HbA1c), and Metabolic Syndrome (MeS) Mediate the Association of the Adherence to the Dietary Approaches to Stopping Hypertension (DASH) and Mediterranean Diet (MeD) With Nonalcoholic Fatty Liver Disease

**DOI:** 10.3389/fnut.2022.921415

**Published:** 2022-07-07

**Authors:** Azam Doustmohammadian, Sakineh Nouri Saeidlou, Saeed Esfandyari, Esmaeel Gholizadeh, Mansooreh Maadi, Nima Motamed, Hossein Ajdarkosh, Mahmoodreza Khoonsari, Cain C. T. Clark, Farhad Zamani

**Affiliations:** ^1^Gastrointestinal and Liver Diseases Research Center, Iran University of Medical Sciences, Tehran, Iran; ^2^Food and Beverages Safety Research Center, Urmia University of Medical Science, Urmia, Iran; ^3^Asadabad School of Medical Sciences, Hamadan, Iran; ^4^Department of Social Medicine, Zanjan University of Medical Sciences, Zanjan, Iran; ^5^Centre for Intelligent Healthcare, Coventry University, Coventry, United Kingdom

**Keywords:** Mediterranean diet, DASH, NAFLD, dietary acid load, HbA1c, metabolic syndrome, structural equation model

## Abstract

The study aimed to investigate the association of adults adhering to Dietary Approaches to Stop Hypertension (DASH) and Mediterranean diet (MeD) with nonalcoholic fatty liver disease (NAFLD) using structural equation modeling (SEM) in Iran. In this population-based cross-sectional study, 3,220 adults (44.65% female) aged ≥18 years were selected from the Amol Cohort Study (AmolCS). The dietary intakes were assessed by a validated 168-item semi-quantitative food-frequency questionnaire (FFQ). Residual method energy adjustment of MeD and DASH scores were calculated. Demographic characteristics and anthropometric and laboratory measurements were collected. NAFLD was diagnosed by an expert radiologist *via* ultrasound sonography. Based on the primary hypothesis, DASH, MeD, and NAFLD were fitted into models. Metabolic syndrome (MeS) as a potential risk factor directly affected NAFLD risk in all these models. In both genders, the higher adherence to DASH negatively affected NAFLD risk indirectly through the two following paths. (1) Dietary acid load (DAL) and metabolic syndrome (2) DAL and hemoglobin A1c (HbA1c). In addition, the higher DAL positively affected NAFLD risk among male participants indirectly *via* increasing HbA1c level and MeS (from DAL to HbA1c: β = 0.07, *P* < 0.001; from HbA1c to MeS: β = 0.10, *P* < 0.001). Similarly, in both genders, the relationship between MeD and NAFLD was mediated through (1) DAL, HbA1c, and MeS and (2) DAL and MeS. Further, among male participants, the MeD and NAFLD risk were also associated *via* the mediators of HbA1c and MeS. In female participants, the higher MeD score was directly associated with a reduction of NAFLD risk (β = −0.07, *P* = 0.008). The present study found three important mediators, including DAL, HbA1c, and MeS, in the association of DASH and MeD scores with NAFLD risk. Preventive and therapeutic interventions should target the mediators, including DAL, HbA1c, MeS, and its components, to reduce NAFLD incidence in the general population.

## Introduction

Worldwide, the prevalence of nonalcoholic fatty liver disease (NAFLD) has increased due to the global increase in overweight and obesity (1, 2). Globally, 25% of the adult population suffers from NAFLD, and its rate was reported to be as high as 40% in some Asian countries (1, 3, 4). The prevalence of NAFLD has been reported with wide variations in different areas of Iran. Indeed, based on a meta-analysis study, the total prevalence of NAFLD was 33.9% in Iran, whilst, in comparison, this rate was reported at 16.47 and 43.8% in Shahrekord (southwest of Iran) and Amol (north of Iran), respectively (5–8).

Nonalcoholic fatty liver disease is diagnosed based on fat accumulation in 5% of hepatocytes by histological assessment or non-invasive imaging (2, 9). NAFLD patients are at higher risk for progression to cirrhosis or directly to Hepatocellular Carcinoma (HCC) (2, 4). NAFLD is a multifactorial disease that involves many risk factors related to lifestyle and diet, including metabolic syndrome (MeS), obesity, type 2 diabetes mellitus, dyslipidemia, and insulin resistance (4, 10). Indeed, several studies have demonstrated that obesity, diabetes, and MeS increase NAFLD risk (11–13).

Nutrition is one of the most critical factors affecting the development of NAFLD. The different dietary patterns and habits can prevent or, conversely, increase the progression of NAFLD (14, 15). The relationship between dietary patterns and NAFLD has been evaluated in several studies (8, 16–18), and it has been shown that the Mediterranean diet (MeD) (19) and Adherence to the Dietary Approaches to Stop Hypertension (DASH) were inversely associated with NAFLD (18, 20–23).

Iranian clinicians recommend MeD as a dietary choice for NAFLD treatment according to the EASL-EASD-EASO Clinical Practice Guidelines, which is effective mainly through reducing insulin resistance and lipid serum concentrations (24). A high intake of plant-based foods such as fruits, vegetables, legumes, nuts and seeds, whole grains, and foods rich in monounsaturated fatty acids (MUFA) characterizes the MeD (25), which is associated with a lower risk of many chronic diseases (26–28). In addition to MeD, the DASH diet, which is a low-glycemic index, low sodium, low-energy-dense dietary pattern rich in phytoestrogens, magnesium, potassium, and dietary fiber (29), has been shown to improve blood pressure and hyperlipidemia, which may also translate to protective effects in NAFLD (30). Despite the apparent benefits of the MeD and DASH diet in lowering the risk of chronic diseases (31, 32), evidence for their effectiveness in NAFLD is scarce (22, 23).

The majority of existing research on this issue has focused on western populations, and not all studies examined these healthy dietary patterns compliance using a uniform scale. However, the relationship between these dietary patterns has not been well investigated in the Middle Eastern population, including Iran, which has different dietary patterns of DASH and MeD components than those in North America and Europe, where the scores were initially developed (20, 33). Previous studies have investigated the relationship between healthy dietary patterns and NAFLD prevalence using the first generation of multivariate techniques, which are limited by performing each analysis separately (34–36). Since the predisposing nutritional, lifestyle, and metabolic factors of NAFLD are closely interrelated, considering the mediators of these associations, rather than merely examining their direct associations, might help us better understand how these variables contribute to the development of NAFLD. Given that there is no approved specific medical therapy for NAFLD, and lifestyle modifications are the cornerstone of treatment for NAFLD, providing a comprehensive model addressing the mechanisms of the effect of dietary patterns on NAFLD to prevent or intervene on NAFLD is needed.

Accordingly, the purpose of the present study was to examine the association of DASH and MeD with NAFLD using structural equation modeling (SEM) while considering the effect of other variables on both dietary patterns and NAFLD among Iranian adults. SEM is increasingly popular, and it is a multivariate analysis technique that enables the measurement of both direct and indirect effects of variables and incorporates models with multiple dependent variables by using several regressions (37).

### Conceptual Framework

**FIGURE 1:**
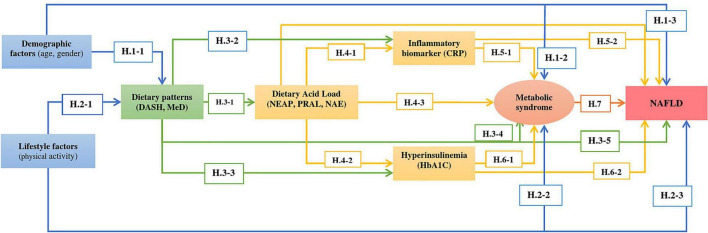
Proposed model of the relationship between dietary patterns (DASH and MeD) and NAFLD considering the effect of other variables on determinants and outcome.

According to the literature review, a conceptual model consisting of baseline predictors, mediators, and the NAFLD outcome was developed (Figure 1).

In this study, the following hypotheses were tested:

Several studies have revealed the association between demographics with dietary patterns (38–40), and according to Mumme et al., females had higher scores of MeD (39). A previous study showed that greater age was positively associated with higher MeD adherence (40) (Hypothesis 1.1).

• Most researchers have reported that MeS prevalence was related to demographic characteristics, including aging (41–44) (Hypothesis 1.2).
• NAFLD was also associated with age and gender and was more prevalent in older people and women (45, 46) (Hypothesis 1.3).
• Healthy lifestyle factors were expected to be related to DASH or MeD patterns and decreased metabolic disorders such as MeS and NAFLD. According to the findings of a study, optimal adherence to the MeD was directly related to education, non-smoking and higher physical activity levels (47, 48) (Hypothesis 2.1).
• Many lifestyle factors affect the occurrence of MeS, and dietary habits and physical activity are the main factors (49). The prevalence of MeS is increasing worldwide due to the increased prevalence of obesity and a sedentary lifestyle (50) (Hypothesis 2.2).
• Increasing obesity due to low physical activity levels and a sedentary lifestyle is an important predictor of NAFLD (51). Lifestyle modification is the primary treatment of NAFLD. Physical activity and MeD have effectively reduced the NAFLD score (52) (Hypothesis 2.3).
• Acid levels are higher in diets that include more animal products than fruits and vegetables (53). Diet acidosis and acid load are estimated using established methods, including net endogenous acid production (NEAP), potential renal acid load (PRAL), endogenous acid excretion (NAE), and dietary acid load (DAL). MeD and DASH contain higher amounts of fruits and vegetables, and it contributes to lower NEAP and PRAL and, therefore, a lower acid load (54). Higher scores of DAL are associated with adverse health outcomes, and adherence to MeD and DASH was associated negatively with DAL indices (54, 55) (Hypothesis 3.1).
• Dietary patterns can affect the expression of inflammatory biomarkers (56), where several studies have shown that MeD and DASH effectively improve circulating serum inflammatory biomarkers, such as C-reactive protein (CRP) (57, 58) (Hypothesis 3.2).
• MeD maybe had a beneficial effect on glycemic factors such as hemoglobin A1c (HbA1c) (59, 60). Research has also observed that DASH improves HbA1c, fasting blood glucose, and HOMA-IR (31) (Hypothesis 3.3).
• DASH and MeD are inversely associated with MeS (61–64) (Hypothesis 3.4).
• Since MeS and NAFLD share multiple risk factors, we expected that DASH/MeD would be associated inversely with NAFLD (18, 23, 65) (Hypothesis 3.5).
• DAL can play a role in metabolic acidosis, leading to inflammation. Based on previous studies, we expected a positive association between DAL, CRP, and HbA1c (66) (Hypothesis 4.1 and Hypothesis 4.2).
• The hypothesis is that higher scores of DAL, NEAP, PRAL, and NAE increase the risk of MeS (55, 67) (Hypothesis 4.3) and are associated with NAFLD (Hypothesis 4.4) (68).
• Inflammatory biomarkers such as CRP are positively associated with MeS (69–71) (Hypothesis 5.1).
• Given this evidence, we hypothesized that CRP level is high in NAFLD patients, and this relationship has been reported in previous studies (72, 73) (Hypothesis 5.2).
• HbA1c level may be used as a marker in identifying MeS (74). It has been reported that HbA1c levels ≥5% is a risk factor for MeS (75) (Hypothesis 6.1).
• We expected that there would be a direct association between HbA1c level and NAFLD (76, 77) (Hypothesis 6.2), and NAFLD patients are also at higher risk for developing MeS (78, 79) (Hypothesis 7).

## Materials and Methods

### Study Population

The current study data were obtained from a large cohort study in Amol city (AmolCS) in the north of Iran. The details of the AmolCS are presented elsewhere (80). Briefly, in AmolCS, a total of 5,147 adults aged ≥18 years in both urban and rural areas were followed up for MeS, cardiovascular disease (CVD), diabetes mellitus, and NAFLD. At the beginning of AmolCS, people who had a history of diseases, including malignancy, thyroid disorder, autoimmune diseases, and physical and mental disability, were excluded.

### Inclusion and Exclusion Criteria

In this cross-sectional study, the study participants were adults of Iranian nationality aged ≥18 years, willing to engage in the study, and longtime inhabitants of Amol. The exclusion criteria were alcohol consumption (>30 g/day for men and >20 g/day for women), viral hepatitis, regular consumption of drug-related steatosis (except for NAFLD), lactation, pregnancy, and following a specific dietary or physical activity regimens.

A total of 829 subjects were eliminated due to missing data in assessing abdominal ultrasonography, food frequency questionnaire (FFQ), and energy intake misreporting. Finally, data from 3,220 participants (1,438 females and 1,782 males) were analyzed.

The ethics committee of the Iran University of Medical Sciences (IUMS) approved the study, and all participants signed written informed consent.

### Demographic Characteristics and Anthropometric Measurements

Demographic characteristics, including age, smoking, physical activity (MET-h/d), alcohol drinking, and having diseases including diabetes, MeS, and CVD, were obtained from the cohort study data. Anthropometric indices [waist circumference (WC), height, and weight] were measured in the participants, and body mass index (BMI) was calculated using the BMI = weight (kg)/height (m^2^) formula. Weight was measured in light clothing and reported to the closest 100 g. WC was measured using tape while standing with feet shoulder-width apart position in the midpoint between the iliac crest and lowest rib. Blood pressures were measured in a sitting position after five minutes of rest. A minimum of two readings at intervals of at least one minute was obtained, and the average of those readings was reported for the blood pressure of patients (81).

### Laboratory Measurements

One blood sample was drawn from participants after eight hours of fasting. Blood samples were incubated, and then they were centrifuged at 3,000 rpm for ten min. Fasting blood sugar (FBS), HbA1c, and lipid profiles, including HDL (high-density lipoprotein), TG (triglycerides), and TC (total cholesterol), were measured. In addition, alanine aminotransferase (ALT), aspartate aminotransferase (AST), gamma-glutamyl transferase (GGT), CRP, hepatitis B virus surface antigens, and hepatitis C virus antibodies, were all assessed.

The A1C level was measured by a Variant machine (Bio-Rad, Hercules, CA, United States). The BS200 Auto analyzer was used to assess laboratory measurements enzymatically according to the manufacturer’s protocol (Mindray, China). The Friedewald equation was used to determine serum low-density lipoprotein cholesterol (LDL) (5). The third-generation ELISA (Enzyme-Linked Immuno-Sorbent Assay) technique was utilized to evaluate hepatitis B viruses (HBV) indicators such as HbsAg, HBsAb, and HBcAb by Acon kits (Acon Laboratory, San Diego, CA, United States).

The Iranian National Reference Laboratory re-evaluated 10% of the blood samples, and all laboratory readings were found to have a variance coefficient of 1.7–3.8%.

### Dietary Assessment

A validated semi-quantitative food-frequency questionnaire (FFQ) was used to evaluate the dietary intakes (82). For each food item on the list, participants were asked about the usual frequency of consumption in a commonly used unit or portion size (daily, weekly, and monthly) over the previous year. The consumption intake of each food item was calculated as gram/day by household measures (83). The food composition table (FCT) of the United States Department of Agriculture (USDA) (84) and Iranian FCT for traditional Iranian foodstuffs (85) were used to determine nutrients and energy consumption.

#### Dietary Approaches to Stopping Hypertension Score

Dietary Approaches to Stopping Hypertension dietary score as a measure of adherence to the DASH dietary pattern was calculated using the typical DASH diet rating initially defined by Fung et al. (29). This method considered quintile intakes of eight components (vegetables, fruits, whole grains, nuts and legumes, low-fat dairy products, salt, red and processed meats, and sweetened drinks). The lowest intake receives one point, and the highest receives five points. The overall score ranges from 8 (the lowest adherence) to 40 (the highest adherence).

#### Mediterranean Diet Score

The degree of adherence to the MeD was assessed based on the Trichopoulou et al. scale (86). Each of the nine components was assigned a value of 0 or 1 on this scale, using the median as the cutoff threshold. Participants whose consumption was below the median for the beneficial components, such as fruits, vegetables, legumes, nuts, cereal, and fish, were given a value of 0, while those whose consumption was at or above the median were given a value of 1. The above coding was inversely applied for dairy products, poultry, red, and processed meats (below the median consumption: value 1, equal or above the median consumption: value 0). Before the score ranking, the adjustment for energy intake using the residual method was made for all food groups.

#### Dietary Acid Load

Three scores, including the NEAP, PRAL score, and the net NAE score, are commonly used to estimate DAL. The intestinal absorption rates for protein, potassium, calcium, magnesium, and phosphate define urine pH in healthy adults estimated by the PRAL score. A positive PRAL or NEAP score reflects an alkaline-forming potential, whereas a negative score is an acid-forming indicator (87, 88).

The following formula calculated net acid excretion (NAE):

1) **Potential renal acid load (PRAL)**

PRAL(mEq/day)=[(0.49×protein[(g/day)]+[(0.037

×phosphorus(mg/day)]-[(0.0211×potassium(mg/day)]

 -[0.0263×magnesium⁢(mg/day)]

-[0.013×calcium⁢(mg/day)]

2) **Organic acids (OA)**

OA⁢(mEq/day)=[body⁢surface⁢area⁢(m2)]× 41⁢(mEq/day⁢per⁢ 1.73⁢m2).

3) **Body surface area (BSA) (89, 90)**

$$\mathrm{Body}\,\mathrm{surface}\,\mathrm{area}\,\left({\text{m}}^{2}\right)=0.007184\times \text{height}\,\left(\text{cm}\right)\,\,0.725\times \text{weight}\,\left(\text{kg}\right)\,0.425$$

4) **DAL proxy (NAE)**
  NAE(mEq/day) = potential renal acid load(PRAL) + organic acids (OA) (87)

#### MeS Definition and Diagnostic

Metabolic syndrome was identified when three of the following five risk factors were present, according to the National Cholesterol Education Program Adult Treatment Panel III (91).

• Fasting blood glucose of more than 100 mg/dl or medication treatment for high blood sugar
• Waist circumference more than 102 cm in men, waist circumference more than 88 cm in women
• Serum TAG more than 150 mg/dl or TAG therapy with medication
• Serum HDL levels of less than 40 mg/dl in men and 50 mg/dl in women, or medication treatment for low HDL levels
• Blood pressure of more than 130/85 mmHg or medication treatment for high blood pressure.

#### Abdominal Ultrasonography

Nonalcoholic fatty liver disease was diagnosed by an expert radiologist using ultrasound sonography. A 3–5 MHz transducer was used to provide sagittal, longitudinal, lateral, and intercostal views. In the same way, blurring of portal or hepatic veins, as well as a significant rise in hepatic echogenicity, were related criteria for fatty liver confirmation.

### Statistical Analysis

Participant characteristics, including age, physical activity, and mediating factors, such as DAL, HbA1c, BMI, CRP, MeS, and its components by gender, were summarized in terms of Mean ± SD for continuous variable and as a percentage (%) for categorical variables.

In the current study, the hypothesized model of the direct and indirect relationship among observed and latent variables was identified and evaluated through the following steps. First, the confirmatory factor analysis (CFA) method was applied to verify the measurement model of MeS and its components by testing the association among the observed variables and their underlying latent construct (s). After that, the SEM statistical approach was applied to test the suggested hypothesis. The model estimation underwent several iterations. There was no multi-collinearity issue (*r* > 0.7 or *r* < −0.7) between variables, conferring on a correlation matrix.

The χ^2^, χ^2^/df, Root Mean Square Error of Approximation (RMSEA), goodness-of-fit index (GFI), adjusted goodness-of-fit index (AGFI), and comparative fit index (CFI) were used to evaluate the absolute fit of the final models to the data. A satisfactory model fit was defined as GFI, AGFI, and CFI values greater than 0.90 and RMSEA values less than 0.08 (92). All data analyses were conducted by SPSS 24.0 (Chicago, IL, United States) and AMOS 24.0 software (IBM Corp., Armonk, NY, United States). The statistical significance level was set, *a priori*, at *P* < 0.05.

## Results

### Baseline Characteristics of the Study Participants

Baseline characteristics by gender are shown in Table 1. The mean age of participants was 46.96 ± 14.67 years, and NAFLD was diagnosed in 44.6% of participants, with no significant difference between the two genders. The prevalence of MeS was significantly higher in females (*P* < 0.001). Significant differences between males and females were observed in all anthropometric, lifestyle, and laboratory variables, except for LDL (*P* = 0.30). Means of all variables were higher in males than females, except for BMI, TC, HDL, FBS, HBA1, and CRP.

**TABLE 1:** Characteristics of the study population by gender in Iranian adults of AmolCS.

Characteristic	All (*n* = 3,220)	Female (*n* = 1,438)	Male (*n* = 1,782)	*P*-value
Age (years)	46.96 (14.67)	45.68 (14.05)	48.00 (15.08)	<0.001
BMI (kg/m^2^)	28.0 (5.0)	29.52 (5.33)	26.78 (4.34)	<0.001
Waist circumference (cm)	88.83 (11.24)	87.90 (12.05)	89.63 (10.48)	<0.001
Past/current smoking (%)	467 (14.5)	8 (0.60)	459 (25.80)	<0.001
Alcohol drinking (%)	189 (5.9)	5 (0.30)	184 (10.30)	<0.001
NAFLD (%)	1,473 (44.6)	637 (44.3)	800 (44.9)	0.73
MeS (%)	866 (26.90)	512 (35.60)	354 (19.90)	<0.001
Physical activity level (%)				0.02
Low (<600)	1,229 (38.20)	585 (40.70)	644 (36.10)	
Moderate (600 to <3,000)	1,984 (61.60)	851 (59.20)	1,133 (63.60)	
High (≥3,000)	7 (0.20)	2 (0.10)	5 (0.30)	
**Laboratory factors (*M* ± SD)**				
TG (mg/dl)	133.58 (90.08)	127.93 (88.34)	138.15 (91.23)	0.001
Total cholesterol (mg/dl)	180.62 (40.33)	183.47 (41.94)	178.31 (38.85)	<0.001
HDL (mg/dl)	43.71 (11.74)	46.10 (11.76)	41.77 (11.36)	<0.001
LDL (mg/dl)	99.15 (26.42)	99.69 (26.72)	98.72 (26.16)	0.30
SBP (mmHg)	114.77 (19.20)	113.51 (20.48)	115.79 (18.04)	0.001
DBP (mmHg)	71.66 (11.81)	70.78 (12.28)	72.36 (11.36)	<0.001
FBS (mg/dl)	105.81 (34.97)	108.45 (40.33)	103.68 (29.79)	<0.001
HbA1c (%)	4.56 (0.91)	4.60 (0.94)	4.53 (0.90)	0.02
ALT (IU/L)	24.04 (18.10)	19.74 (13.89)	27.50 (20.23)	<0.001
AST (IU/L)	21.60 (10.39)	19.44 (8.22)	23.35 (11.56)	<0.001
GGT (IU/L)	27.01 (19.02)	24.04 (18.54)	29.42 (19.07)	<0.001
ALKP (IU/L)	197.79 (59.89)	195.87 (67.37)	199.34 (53.05)	0.10
CRP	1.60 (0.70-4.00)	2.00 (1.00-5.00)	1.10 (0.50–3.00)	<0.001
**Dietary factors (*M* ± SD)**				
Energy intake (kcal/day)	2,329.99 (664.12)	2,171.07 (599.92)	2,458.24 (658.51)	<0.001
DAL (mEq/day)	645.84 (143.20)	596.39 (130.47)	693.57 (150.78)	<0.001

*Data are presented as mean (SD) for quantitative variables, median (25–75 percentile) for CRP, and n (%) for qualitative variables.*

### Confirmatory Factor Analysis

**FIGURE 2:**
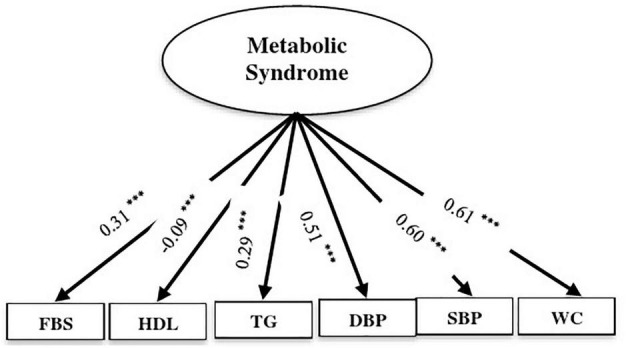
Confirmatory factor analysis (CFA) of the measurement model of metabolic syndrome components. Ellipses represent latent variables; boxes represent observed variables. All coefficients are standardized and have a *P*-value < 0.001. Fit indices: CMIND/DF = 13.64, DF = 4, GFI = 0.99, AGFI = 0.97, CFI = 0.99, IFI = 0.99, SRMR = 0.02, RMSEA = 0.06 (^***^ *P* < 0.001).

Based on the CFA, the constructs of MeS components had an acceptable fit index (χ^2^/df = 17.94, GFI = 0.98, AGFI = 0.96, CFI = 0.97, IFI = 0.97, SRMR = 0.03, RMSEA = 0.07). All components were significantly related to MeS (*P* < 0.001). Waist circumstance and systolic blood pressure explained the highest proportion of the variance of MeS (Figure 2).

### The Link Between Dietary Patterns and Nonalcoholic Fatty Liver Disease Using Structural Equation Modeling

**FIGURE 3:**
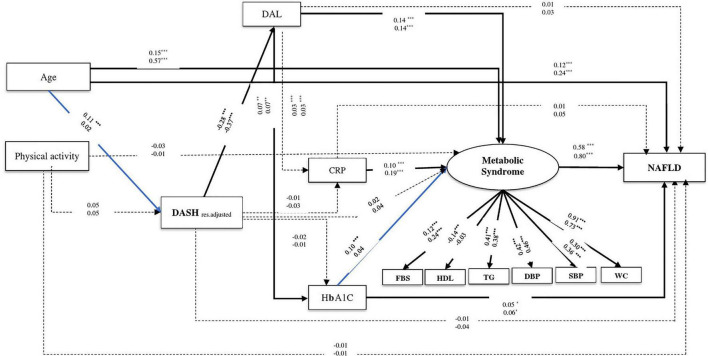
The final structural model of the relationship between residual adjusted DASH and NAFLD. The model fit indices: χ^2^/df = 5.21, *P* < 0.001, GFI = 0.98, AGFI = 0.96, CFI = 0.95, IFI = 0.95, SRMR = 0.03, RMSEA = 0.03. The values on the paths represent standardized regression coefficients. The upper-faced numbers refer to men, whereas the numerical values below them refer to women. Arrows in bold represent statistically significant associations (*P < 0.05; ^**^P < 0.01; ^***^P < 0.001). Blue arrows refer to males.

**FIGURE 4:**
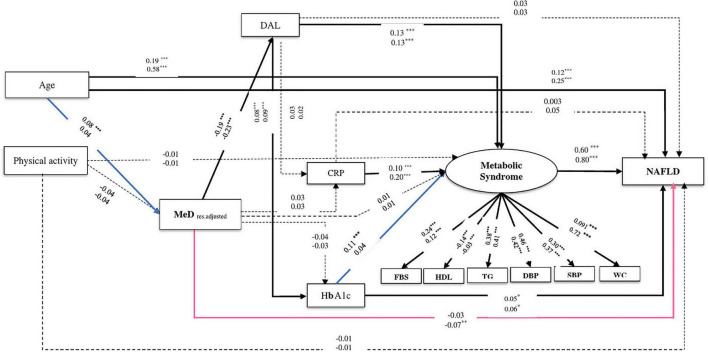
The final structural model of the relationship between residual adjusted MeD and NAFLD. The model fit indices: χ^2^/df = 5.21, *p* < 0.001, GFI = 0.98, AGFI = 0.96, CFI = 0.95, IFI = 0.95, SRMR = 0.03, RMSEA = 0.03. The values on the paths represent standardized regression coefficients. The upper-faced numbers refer to males, whereas the numerical values below them refer to females. Arrows in bold represent statistically significant associations (*P < 0.05; ^**^P < 0.01; ^***^P < 0.001). Pink arrows refer to females whereas blue arrows refer to males.

Dietary Approaches to Stopping Hypertension, MeD, and NAFLD were fitted in models based on the primary hypothesis (Figures 3, 4). MeS, as a potential risk factor, directly affected NAFLD in all models. Age was a demographic factor that positively affected MeS and NAFLD. The prevalence of MeS and NAFLD was higher in older adults. In both genders, the higher score of the DASH diet was negatively related to NAFLD risk indirectly through the two following paths. (1) DAL (from DASH to DAL, in males β = −0.28, *P* < 0.001; in females β = −0.37, *P* < 0.001), and MeS (for both gender from DAL to MeS: β = 0.14, *P* < 0.001; from MeS to NAFLD, in male β = 0.58, *P* < 0.001; in female β = 0.80, *P* < 0.001), (2) DAL and HbA1c (from DAL to HbA1c, in both genders: β = 0.07, *P* < 0.001; from HbA1c to NAFLD, in males: β = 0.05, *P* = 0.03; in females: β = 0.06, *P* = 0.03). In addition, the higher DAL positively affected NAFLD risk among male participants indirectly *via* increasing HbA1c level and MeS (from HbA1c to MeS: β = 0.10, *P* < 0.001).

Similarly in both genders, the relationship between MeD and NAFLD was mediated by two pathways, including (1) through the mediators of DAL, HbA1c, and MeS (of MeD to DAL, in males: β = −0.19, *P* < 0.001, in females: β = −0.23, *P* < 0.001; of DAL to HbA1c, in males: β = 0.08, *P* < 0.001, in females: β = 0.09, *P* < 0.001; of HbA1c to NAFLD, in males: β = 0.05, *P* = 0.02, in females: β = 0.06, *P* = 0.02) and (2) through the mediators of DAL and MeS (of DAL to MeS, in both genders: β = 0.13, *P* < 0.001; of MeS to NAFLD, in males: β = 0.60, *P* < 0.001, in females: β = 0.80, *P* < 0.001). Further, among male participants, the association of MeD and NAFLD was also mediated *via* HbA1c and MeS (of HbA1c to MeS, β = 0.11, *P* < 0.001). In female participants, the higher MeD score was directly associated with a reduction of NAFLD risk (β = −0.07, *P* = 0.008) (see Table 2 and Figures 3, 4).

**TABLE 2:** Standardized total effect, direct effect, and indirect effect of the association of DASH and MeD with NAFLD in Iranian adults of AmolCS.

From	To	Direct effect	Indirect effect	Total effect
**DASH structural equation model (model 1)**				
**Male**				
DASH	DAL	−0.276	–	−0.276***
DAL	HbA1c	0.071	–	0.071**
	MeS	0.140	0.010	0.150***
CRP	MeS	0.099	–	0.099***
HbA1c	MeS	0.103	–	0.103*
	NAFLD	0.046	0.059	0.105*
MeS	NAFLD	0.576	–	0.576***
**Female**				
DASH	DAL	−0.370	–	−0.37***
DAL	HbA1c	0.075	–	0.075**
	MeS	0.144	0.010	0.154***
CRP	MeS	0.192	–	0.192***
HbA1c	NAFLD	0.056	0.037	0.093*
MeS	NAFLD	0.799	–	0.799***
**MeD structural equation model (model 2)**				
**Male**				
MeD	DAL	–0.189	–	–0.189***
	HbA1c	0.059	–0.015	0.044*
DAL	HbA1c	0.078	–	0.078***
	MeS	0.133	0.010	0.143***
CRP	MeS	0.098	–	0.099***
HbA1c	MeS	0.106	–	0.106***
	NAFLD	0.047	0.060	0.107*
MeS	NAFLD	0.577	–	0.577***
**Female**				
MeD	DAL	–0.233	–	–0.233***
	NAFLD	–0.067	0.003	–0.067**
DAL	HbA1c	0.092	–	0.092***
	MeS	0.133	0.010	0.143***
CRP	MeS	0.196	–	0.196***
HbA1c	NAFLD	0.059	0.036	0.095*
MeS	NAFLD	0.804		0.804***

*p < 0.05, **p < 0.01, ***p < 0.001.

## Discussion

To our knowledge, this is the first study to have examined the association between adherence to healthy dietary patterns and the prevalence of NAFLD in a large-scale general population using the SEM approach. The main advantage of the SEM method is its ability to explore complex correlations among variables using a systematic approach while also managing measurement errors (93).

The findings of the current study suggested that DAL, HbA1c, and MeS were the main mediators of the association between low adherence to healthy diets (DASH and MeD) and the high prevalence of NAFLD. Previous studies have discussed the relationship between the components of MeS and the risk of developing NAFLD (94). Indeed, in a meta-analysis, Sookoian and Pirola (95) explored 15 well-structured studies and concluded that the components of MeS were strong predictors of NAFLD. Our findings were concordant with previous studies suggesting that MeS remains the most potent factor related to NAFLD risk (96).

In our study, adherence to DASH was indirectly (through the negative effect on DAL and MeS) associated with a lower prevalence of NAFLD. Several mechanisms may explain the health benefits of the DASH dietary pattern. High consumption of vegetables, fruits, whole grains, and unsaturated vegetable oils characterizes this diet. High dietary fiber consumption attenuates metabolic abnormalities, including TG and abdominal obesity, by delaying stomach emptying (97). Calcium, another crucial DASH component, protects against MeS by lowering adiposity (98). It is also crucial to reduce fat absorption by forming insoluble fatty acid soaps in the gut. Therefore, calcium plays a critical function in WC reduction (98). Moreover, it is low (≤5 serves of sweets and added sugars per week for a 2,000-calorie diet) in sugar-sweetened drinks, added sugars, and red and processed meats (99). As a result, this diet improves glycemic and lipid metabolism and aids weight loss while lowering CVDs. All of these outcomes are primary goals of NAFLD treatment; hence, this diet has attracted the attention of NAFLD specialists. In a randomized controlled trial of 60 obese adults with NAFLD and elevated serum ALT levels, the results revealed that consumption of the DASH diet for eight weeks decreased BMI and steatosis severity, as well as improved aminotransferases and metabolic markers, including insulin, HOMA index, serum TG, and the total to HDL cholesterol ratio compared to control group (97). Other studies among the Chinese general population have reported an inverse association between adherence to DASH and risk of NAFLD (with 22 and 18% low risk of NAFLD) (22, 35).

On the other hand, the DASH-type dietary pattern as an alkali-rich diet containing high amounts of alkalizing minerals, potassium, calcium, and magnesium could explain the DAL lowering effect of this diet (100, 101). Indeed, adherence to the MeD pattern in both genders was indirectly associated with the risk of NAFLD in this population through the negative effect on DAL and MeS. The MeD is characterized by high consumption of legumes, grains, vegetables, fresh fruits, olive oil, and nuts. Fish and white meat are consumed in moderation in this diet, and the consumption of red and processed meats and high-fat dairy products are limited. The MeD is also characterized by a low ratio of saturated fatty acids (SFAs) and cholesterol (9% total energy intake); whilst, in contrast, mono-unsaturated fatty acids (MUFAs) and poly-unsaturated fatty acids (PUFAs) are high (19% total energy intake from MUFA, and 5% total energy intake from PUFA), with a favorable ratio of omega-3 to omega-6 (102).

Several studies have reported that dietary patterns high in omega-3 PUFAs can lower insulin resistance and intrahepatic TG levels, resulting in steatohepatitis improvement (102).

Furthermore, PUFAs have been shown to help prevent cardiovascular events by improving insulin sensitivity, functioning as an anti-inflammatory, and lowering oxidative stress (7).

The MeD also has a high content (55–60% of the total energy intake) of complex carbs and fibers, which helps to prevent fatty liver disease. In epidemiological studies, refined sugar, particularly high-fructose corn syrup, has been linked to NAFLD risk (103). Furthermore, the MeD is high in whole grains, which includes a lot of fiber, which may be advantageous to NAFLD patients for various reasons. The first is that regulation of gut microbiota through their prebiotic actions may be improved, which is known to play a role in the development and progression of NAFLD. Second, whole grain consumption can reduce the risk of dyslipidemia, CVD, and diabetes (37, 80, 104). Another feature of the MeD is low consumption of red and processed meat, including SFAs and cholesterol, which has been linked to NAFLD development in case-control and cross-sectional studies (11, 105).

The content of vegetables and fruits in MeD are sources of potassium salts of organic acids, which form bicarbonate; thus, MeD is net-base-yielding and can reduce DAL (106). Observational studies reveal that a low DAL may help to reduce the incidence of obesity, MeS, diabetes, hypertension, CVDs, and mortality (107–110). The underlying mechanisms that link DAL to NAFLD may be related to the Insulin Resistance (IR) associated with acidic diets. The consequent hyperglycemia appears to increase the inflammation and IR in the liver, which may cause NAFLD to increase available free fatty acids (110). In Asian adults, Chan et al., in a study conducted on 793 subjects, reported a positive association between estimated diet-induced acid load *via* NEAP with an increased likelihood of NAFLD (111).

Although we present a novel addition to the literature, our study also has several limitations. First, the conclusions of this study may not apply to other countries, particularly the western population, given that the study sample was drawn only from the Iranian people. Because different countries have diverse eating cultures, more research is needed to ascertain whether the mediation model proposed in this study can be applied to other countries with different dietary patterns. Second, because we lacked data on socioeconomic factors such as education level, job position, income, and ethnicity information (112) as well as OCP (113) and supplement (114) usage, some confounders may not have been captured and adjusted in the proposed mediation model. As a result, considering the potential influence of such uncontrolled confounders, the results should be interpreted with caution. Nonetheless, the findings of the current study can be used to offer baseline data and/or markers for future studies to control confounding variables. Third, the FFQ is a dietary evaluation instrument with a strong recall bias. Despite this, FFQs are still the most common dietary assessment technique in cohort studies. Finally, the cross-sectional nature of the present study precludes making inferences regarding the directionality of the association, resulting in a high risk of reverse-causality bias.

The causal links between mediators and NAFLD are unclear because the mediators and follow-up NAFLD status were collected at the same time. As a result, more research is needed to determine whether strategies for addressing mediated disorders can also treat NAFLD problems among the general population.

## Conclusion

The present study found three important mediators, including DAL, HbA1c, and MeS, in associating current dietary patterns and NAFLD. Based on our findings, adherence to DASH and MeD should be emphasized in programs attempting to elicit changes in lifestyle for subjects with NAFLD. This study suggests that preventive and therapeutic interventions should target the mediators, DAL, HbA1c, MeS, and their components, to reduce NAFLD incidence in the general population.

## Data Availability Statement

The data analyzed in this study is subject to the following licenses/restrictions: the datasets used and analyzed during the current study are available from the corresponding author on reasonable request. Requests to access these datasets should be directed to FZ, zamani.farhad@gmail.com.

## Ethics Statement

The current study was conducted according to the guidelines in the Declaration of Helsinki, and procedures involving human subjects/patients were approved by the Iran University of Medical Sciences (IUMS) Ethical Committee (No. IR.IUMS.REC.1399.1393). Written informed consent was obtained from all participants before the study. The patients/participants provided their written informed consent to participate in this study.

## Author Contributions

FZ, NM, HA, MK, and AD were responsible for the study concept and design. AD and NM had full access to all data and took responsibility for the integrity of the data and the accuracy of the data analysis. MM and EG were involved in data collection. AD, NM, CC, and SE analyzed and interpreted the data. AD and SN wrote the initial draft of the manuscript. All authors revised the manuscript critically for important intellectual content and approved the final manuscript. FZ was the guarantor and takes responsibility for the manuscript as a whole.

## Conflict of Interest

The authors declare that the research was conducted in the absence of any commercial or financial relationships that could be construed as a potential conflict of interest.

## Publisher’s Note

All claims expressed in this article are solely those of the authors and do not necessarily represent those of their affiliated organizations, or those of the publisher, the editors and the reviewers. Any product that may be evaluated in this article, or claim that may be made by its manufacturer, is not guaranteed or endorsed by the publisher.

## References

[1] Muthiah MD Cheng Han N Sanyal AJ. A clinical overview of non-alcoholic fatty liver disease: a guide to diagnosis, the clinical features, and complications—what the non-specialist needs to know. *Diabetes Obes Metab.* (2022) 24:3–14. 10.1111/dom.14521 34387409

[2] Bence KK Birnbaum MJ. Metabolic drivers of non-alcoholic fatty liver disease. *Mol Metab.* (2021) 50:101143. 33346069 10.1016/j.molmet.2020.101143 PMC8324696

[3] Byrne CD Targher G. Non-alcoholic fatty liver disease-related risk of cardiovascular disease and other cardiac complications. *Diabetes Obes Metab.* (2021) 24:28–43. 10.1111/dom.14484 34324263

[4] Bellentani S. The epidemiology of non-alcoholic fatty liver disease. *Liver Int.* (2017) 37:81–4. 28052624 10.1111/liv.13299

[5] Moghaddasifar I Lankarani K Moosazadeh M Afshari M Ghaemi A Aliramezany M Prevalence of non-alcoholic fatty liver disease and its related factors in Iran. *Int J Organ Transpl Med.* (2016) 7:149. PMC5054138 27721961

[6] Zarean E Goujani R Rahimian G Ahamdi A. Prevalence and risk factors of non-alcoholic fatty liver disease in Southwest Iran: a population-based case-control study. *Clin Exp Hepatol.* (2019) 5:224. 10.5114/ceh.2019.87635 31598559 PMC6781826

[7] Amirkalali B Poustchi H Keyvani H Khansari MR Ajdarkosh H Maadi M Prevalence of non-alcoholic fatty liver disease and its predictors in North of Iran. *Iran J Public Health.* (2014) 43:1275. 26175982 PMC4500430

[8] Salehi-Sahlabadi A Sadat S Beigrezaei S Pourmasomi M Feizi A Ghiasvand R Dietary patterns and risk of non-alcoholic fatty liver disease. *BMC Gastroenterol.* (2021) 21:41. 10.1186/s12876-021-01612-z 33509112 PMC7844966

[9] Neuschwander-Tetri BA. Non-alcoholic fatty liver disease. *BMC Med.* (2017) 15:45. 10.1186/s12916-017-0806-8 28241825 PMC5330146

[10] Finelli C Tarantino G. Non-alcoholic fatty liver disease, diet and gut microbiota. *EXCLI J.* (2014) 13:461. 26417275 PMC4464355

[11] Tomah S Hamdy O Abuelmagd MM Hassan AH Alkhouri N Al-Badri MR Prevalence of and risk factors for non-alcoholic fatty liver disease (NAFLD) and fibrosis among young adults in Egypt. *BMJ Open Gastroenterol.* (2021) 8:e000780. 10.1136/bmjgast-2021-000780 PMC8493908 34610926

[12] Sae-Wong J Chaopathomkul B Phewplung T Chaijitraruch N Sahakitrungruang T. The prevalence of nonalcoholic fatty liver disease and its risk factors in children and young adults with type 1 diabetes mellitus. *J Pediatr.* (2021) 230:e1. 10.1016/j.jpeds.2020.10.043 33250172

[13] Kanwal S Ghaffar T Aamir AH Usman K. Frequency of non-alcoholic fatty liver disease in patients with type-2 diabetes mellitus and its associated risk factors. *Pak J Med Sci.* (2021) 37:1335–41. 10.12669/pjms.37.5.4211 34475908 PMC8377887

[14] Vancells Lujan P Viñas Esmel E Sacanella Meseguer E. Overview of non-alcoholic fatty liver disease (NAFLD) and the role of sugary food consumption and other dietary components in its development. *Nutrients.* (2021) 13:1442. 10.3390/nu13051442 33923255 PMC8145877

[15] Berná G Romero-Gomez M. The role of nutrition in non-alcoholic fatty liver disease: pathophysiology and management. *Liver Int.* (2020) 40:102–8. 32077594 10.1111/liv.14360

[16] Fakhoury-Sayegh N Younes H Heraoui G Sayegh R. Nutritional profile and dietary patterns of Lebanese non-alcoholic fatty liver disease patients: a case-control study. *Nutrients.* (2017) 9:1245. 10.3390/nu9111245 PMC5707717 29135945

[17] Xia Y Zhang Q Liu L Meng G Wu H Bao X Intermediary effect of inflammation on the association between dietary patterns and non-alcoholic fatty liver disease. *Nutrition.* (2020) 71:110562. 10.1016/j.nut.2019.110562 31809956

[18] Zelber-Sagi S Salomone F Mlynarsky L. The Mediterranean dietary pattern as the diet of choice for non-alcoholic fatty liver disease: evidence and plausible mechanisms. *Liver Int.* (2017) 37:936–49. 10.1111/liv.13435 28371239

[19] Ballestri S Nascimbeni F Baldelli E Marrazzo A Romagnoli D Lonardo A. NAFLD as a sexual dimorphic disease: role of gender and reproductive status in the development and progression of nonalcoholic fatty liver disease and inherent cardiovascular risk. *Adv Ther.* (2017) 34:1291–326. 10.1007/s12325-017-0556-1 28526997 PMC5487879

[20] Hassani Zadeh S Mansoori A Hosseinzadeh M. Relationship between dietary patterns and non-alcoholic fatty liver disease: a systematic review and meta-analysis. *J Gastroenterol Hepatol.* (2021) 36:1470–8. 10.1111/jgh.15363 33269500

[21] Vancells Lujan P Vinas Esmel E Sacanella Meseguer E. Overview of non-alcoholic fatty liver disease (NAFLD) and the role of sugary food consumption and other dietary components in its development. *Nutrients.* (2021) 13:1442. 33923255 10.3390/nu13051442 PMC8145877

[22] Xiao ML Lin JS Li YH Liu M Deng YY Wang CY Adherence to the dietary approaches to stop hypertension (DASH) diet is associated with lower presence of non-alcoholic fatty liver disease in middle-aged and elderly adults. *Public Health Nutr.* (2020) 23:674–82. 10.1017/S1368980019002568 31566148 PMC10200450

[23] Hekmatdoost A Shamsipour A Meibodi M Gheibizadeh N Eslamparast T Poustchi H. Adherence to the dietary approaches to stop hypertension (DASH) and risk of nonalcoholic fatty liver disease. *Int J Food Sci Nutr.* (2016) 67:1024–9. 10.1080/09637486.2016.1210101 27436528

[24] Plaz Torres MC Aghemo A Lleo A Bodini G Furnari M Marabotto E Mediterranean diet and NAFLD: what we know and questions that still need to be answered. *Nutrients.* (2019) 11:2971. 10.3390/nu11122971 31817398 PMC6949938

[25] Trichopoulou A Martínez-González MA Tong TY Forouhi NG Khandelwal S Prabhakaran D Definitions and potential health benefits of the Mediterranean diet: views from experts around the world. *BMC Med.* (2014) 12:112. 10.1186/1741-7015-12-112 25055810 PMC4222885

[26] Panico S Mattiello A Panico C Chiodini P. Mediterranean dietary pattern and chronic diseases. *Adv Nutr Cancer.* (2014) 159:69–81. 10.1007/978-3-642-38007-5_5 24114475

[27] Martinez-Lacoba R Pardo-Garcia I Amo-Saus E Escribano-Sotos F. Mediterranean diet and health outcomes: a systematic meta-review. *Eur J Public Health.* (2018) 28:955–61. 10.1093/eurpub/cky113 29992229

[28] Papadaki A Nolen-Doerr E Mantzoros CS. The effect of the Mediterranean diet on metabolic health: a systematic review and meta-analysis of controlled trials in adults. *Nutrients.* (2020) 12:3342. 10.3390/nu12113342 33143083 PMC7692768

[29] Fung TT Chiuve SE McCullough ML Rexrode KM Logroscino G Hu FB. Adherence to a DASH-style diet and risk of coronary heart disease and stroke in women. *Arch Intern Med.* (2008) 168:713–20. 10.1001/archinte.168.7.713 18413553

[30] Filippou CD Tsioufis CP Thomopoulos CG Mihas CC Dimitriadis KS Sotiropoulou LI Dietary approaches to stop hypertension (DASH) diet and blood pressure reduction in adults with and without hypertension: a systematic review and meta-analysis of randomized controlled trials. *Adv Nutr.* (2020) 11:1150–60. 32330233 10.1093/advances/nmaa041 PMC7490167

[31] Chiavaroli L Viguiliouk E Nishi SK Blanco Mejia S Rahelić D Kahleová H DASH dietary pattern and cardiometabolic outcomes: an umbrella review of systematic reviews and meta-analyses. *Nutrients.* (2019) 11:338. 10.3390/nu11020338 30764511 PMC6413235

[32] De la Iglesia R Loria-Kohen V Zulet MA Martinez JA Reglero G Ramirez de Molina A. Dietary strategies implicated in the prevention and treatment of metabolic syndrome. *Int J Mol Sci.* (2016) 17:1877. 10.3390/ijms17111877 PMC5133877 27834920

[33] Mirmiran P Moslehi N Mahmoudof H Sadeghi M Azizi F. A longitudinal study of adherence to the Mediterranean dietary pattern and metabolic syndrome in a Non-Mediterranean population. *Int J Endocrinol Metab.* (2015) 13:e26128. 10.5812/ijem.26128v2 26425127 PMC4584365

[34] Doustmohammadian A Clark CC Maadi M Motamed N Sobhrakhshankhah E Ajdarkosh H Favorable association between Mediterranean diet (MeD) and DASH with NAFLD among Iranian adults of the Amol cohort study (Amolcs). *Sci Rep.* (2022) 12:1–9. 10.1038/s41598-022-06035-8 35136128 PMC8825797

[35] Sun Y Chen S Zhao X Wang Y Lan Y Jiang X Adherence to the dietary approaches to stop hypertension diet and non-alcoholic fatty liver disease. *Liver Int.* (2022) 42:809–19. 34990079 10.1111/liv.15156

[36] Baratta F Pastori D Polimeni L Bucci T Ceci F Calabrese C Adherence to Mediterranean diet and non-alcoholic fatty liver disease: effect on insulin resistance. *Off J Am Coll Gastroenterol.* (2017) 112:1832–9. 10.1038/ajg.2017.371 29063908

[37] Rosato V Temple NJ La Vecchia C Castellan G Tavani A Guercio V. Mediterranean diet and cardiovascular disease: a systematic review and meta-analysis of observational studies. *Eur J Nutr.* (2019) 58:173–91. 10.1007/s00394-017-1582-0 29177567

[38] Naska A Fouskakis D Oikonomou E Almeida M Berg M Gedrich K Dietary patterns and their socio-demographic determinants in 10 European countries: data from the DAFNE databank. *Eur J Clin Nutr.* (2006) 60:181–90. 10.1038/sj.ejcn.1602284 16278696

[39] Mumme K Conlon C von Hurst P Jones B Stonehouse W Heath A-LM Dietary patterns, their nutrients, and associations with socio-demographic and lifestyle factors in older New Zealand adults. *Nutrients.* (2020) 12:3425. 10.3390/nu12113425 33171602 PMC7695209

[40] Khaled K Hundley V Almilaji O Koeppen M Tsofliou F. A priori and a posteriori dietary patterns in women of childbearing age in the UK. *Nutrients.* (2020) 12:2921. 10.3390/nu12102921 32987718 PMC7598658

[41] Kim JH Kim HL Battushig B Yoo JY. Relationship between socio-demographics, body composition, emotional state, and social support on metabolic syndrome risk among adults in rural Mongolia. *PLoS One.* (2021) 16:e0254141. 10.1371/journal.pone.0254141 34570786 PMC8475977

[42] Sy RG Llanes EJB Reganit PFM Castillo-Carandang N Punzalan FER Sison OT Socio-demographic factors and the prevalence of metabolic syndrome among Filipinos from the LIFECARE cohort. *J Atheroscler Thromb.* (2014) 21(Suppl. 1):S9–17. 10.5551/jat.21_sup.1-s9 24452117

[43] Pathania D Bunger R Mishra P Pathak R Arora A. A study to assess prevalence of metabolic syndrome and its socio demographic risk factors in rural area of district Ambala, Haryana. *J Community Med Health Educ.* (2013) 3:226.

[44] Okube OT Kimani ST Mirie W. Gender differences in the pattern of socio-demographics relevant to metabolic syndrome among Kenyan adults with central obesity at a mission hospital in Nairobi, Kenya. *High Blood Press Cardiovasc Prev.* (2020) 27:61–82. 10.1007/s40292-020-00360-7 31981085

[45] Zou B Yeo Y Nguyen V Cheung R Ingelsson E Nguyen M. Prevalence, characteristics and mortality outcomes of obese, nonobese and lean NAFLD in the United States, 1999–2016. *J Intern Med.* (2020) 288:139–51. 10.1111/joim.13069 32319718

[46] Summart U Thinkhamrop B Chamadol N Khuntikeo N Songthamwat M Kim CS. Gender differences in the prevalence of nonalcoholic fatty liver disease in the northeast of Thailand: a population-based cross-sectional study. *F1000Research.* (2017) 6:1630. 10.12688/f1000research.12417.2 29093809 PMC5645706

[47] Marventano S Godos J Platania A Galvano F Mistretta A Grosso G. Mediterranean diet adherence in the Mediterranean healthy eating, aging and lifestyle (MEAL) study cohort. *Int J Food Sci Nutr.* (2018) 69:100–7. 28562120 10.1080/09637486.2017.1332170

[48] del Mar Bibiloni M Julibert A Argelich E Aparicio-Ugarriza R Palacios G Pons A Western and Mediterranean dietary patterns and physical activity and fitness among Spanish older adults. *Nutrients.* (2017) 9:704. 10.3390/nu9070704 28684703 PMC5537819

[49] Takahara M Shimomura I. Metabolic syndrome and lifestyle modification. *Rev Endocr Metab Disord.* (2014) 15:317–27. 25263290 10.1007/s11154-014-9294-8

[50] Yamaoka K Tango T. Effects of lifestyle modification on metabolic syndrome: a systematic review and meta-analysis. *BMC Med.* (2012) 10:138. 10.1186/1741-7015-10-138 23151238 PMC3523078

[51] Romero-Gómez M Zelber-Sagi S Trenell M. Treatment of NAFLD with diet, physical activity and exercise. *J Hepatol.* (2017) 67:829–46. 28545937 10.1016/j.jhep.2017.05.016

[52] Franco I Bianco A Mirizzi A Campanella A Bonfiglio C Sorino P Physical activity and low glycemic index Mediterranean diet: main and modification effects on NAFLD score. Results from a randomized clinical trial. *Nutrients.* (2021) 13:66. 10.3390/nu13010066 33379253 PMC7823843

[53] Frassetto L Banerjee T Powe N Sebastian A. Acid balance, dietary acid load, and bone effects—a controversial subject. *Nutrients.* (2018) 10:517. 10.3390/nu10040517 29690515 PMC5946302

[54] Fatahi S Azadbakht L. Association between dietary acid load with alternative Mediterranean diet and dietary approaches to stop hypertension among Tehranian women. *J Fasa Univ Med Sci.* (2019) 8:1036–45.

[55] Mozaffari H Siassi F Guilani B Askari M Azadbakht L. Association of dietary acid-base load and psychological disorders among Iranian women: a cross-sectional study. *Complement Ther Med.* (2020) 53:102503. 10.1016/j.ctim.2020.102503 33066849

[56] Silveira BKS Oliveira TMS Andrade PA Hermsdorff HHM Rosa COB Franceschini SDCC. Dietary pattern and macronutrients profile on the variation of inflammatory biomarkers: scientific update. *Cardiol Res Pract.* (2018) 2018:4762575. 29725543 10.1155/2018/4762575 PMC5872610

[57] Soltani S Chitsazi MJ Salehi-Abargouei A. The effect of dietary approaches to stop hypertension (DASH) on serum inflammatory markers: a systematic review and meta-analysis of randomized trials. *Clin Nutr.* (2018) 37:542–50. 10.1016/j.clnu.2017.02.018 28302405

[58] Lahoz C Castillo E Mostaza JM De Dios O Salinero-Fort MA González-Alegre T Relationship of the adherence to a Mediterranean diet and its main components with CRP levels in the Spanish population. *Nutrients.* (2018) 10:379. 10.3390/nu10030379 29558396 PMC5872797

[59] Zhong VW Lamichhane AP Crandell JL Couch SC Liese AD The NS Association of adherence to a Mediterranean diet with glycemic control and cardiovascular risk factors in youth with type I diabetes: the search nutrition ancillary study. *Eur J Clin Nutr.* (2016) 70:802–7. 10.1038/ejcn.2016.8 26908421 PMC4935596

[60] Zahedi M Akhlagh SA Aboomardani M Alipoor R Hosseini SA Shahmirzadi AR. Efficacy of Mediterranean diet on blood biochemical factors in type II diabetic patients: a randomized controlled trial. *GMJ.* (2020) 31:714–8.

[61] Asghari G Yuzbashian E Mirmiran P Hooshmand F Najafi R Azizi F. Dietary approaches to stop hypertension (DASH) dietary pattern is associated with reduced incidence of metabolic syndrome in children and adolescents. *J Pediatr.* (2016) 174:178–84.e1. 27156186 10.1016/j.jpeds.2016.03.077

[62] Joyce BT Wu D Hou L Dai Q Castaneda SF Gallo LC DASH diet and prevalent metabolic syndrome in the hispanic community health study/study of Latinos. *Prev Med Rep.* (2019) 15:100950. 10.1016/j.pmedr.2019.100950 31367513 PMC6657306

[63] Ghorabi S Salari-Moghaddam A Daneshzad E Sadeghi O Azadbakht L Djafarian K. Association between the DASH diet and metabolic syndrome components in Iranian adults. *Diabetes Metab Syndr Clin Res Rev.* (2019) 13:1699–704. 10.1016/j.dsx.2019.03.039 31235081

[64] Hassani Zadeh S Salehi-Abargouei A Mirzaei M Nadjarzadeh A Hosseinzadeh M. The association between dietary approaches to stop hypertension diet and Mediterranean diet with metabolic syndrome in a large sample of Iranian adults: Yahs and Tamyz studies. *Food Sci Nutr.* (2021) 9:3932–41. 10.1002/fsn3.2387 34262749 PMC8269560

[65] Trovato FM Castrogiovanni P Malatino L Musumeci G. Nonalcoholic fatty liver disease (NAFLD) prevention: role of Mediterranean diet and physical activity. *Hepatobiliary Surg Nutr.* (2019) 8:167. 31098370 10.21037/hbsn.2018.12.05 PMC6503241

[66] Wu T Seaver P Lemus H Hollenbach K Wang E Pierce JP. Associations between dietary acid load and biomarkers of inflammation and hyperglycemia in breast cancer survivors. *Nutrients.* (2019) 11:1913. 10.3390/nu11081913 31443226 PMC6723571

[67] Jafari A Ghanbari M Shahinfar H Bellissimo N Azadbakht L. The association between dietary acid load with cardiometabolic risk factors and inflammatory markers amongst elderly men: a cross-sectional study. *Int J Clin Pract.* (2021) 75:e14109. 10.1111/ijcp.14109 33624383

[68] Rahbarinejad P Mohamadi Narab M. The association of dietary acid load with non-alcoholic fatty liver disease among iranian adults. *Food and Health*. (2020) 3:25–28.

[69] Dallmeier D Larson MG Vasan RS Keaney JF Fontes JD Meigs JB Metabolic syndrome and inflammatory biomarkers: a community-based cross-sectional study at the Framingham heart study. *Diabetol Metab Syndr.* (2012) 4:1–7. 10.1186/1758-5996-4-28 22716219 PMC3547735

[70] de F Rocha AR de S Morais N Priore SE do CC Franceschini S. Inflammatory biomarkers and components of metabolic syndrome in adolescents: a systematic review. *Inflammation.* (2022) 45:14–30. 10.1007/s10753-021-01549-1 34546513

[71] Shahrokh S Heydarian P Ahmadi F Saddadi F Razeghi E. Association of inflammatory biomarkers with metabolic syndrome in hemodialysis patients. *Renal Fail.* (2012) 34:1109–13. 10.3109/0886022X.2012.713280 22889096

[72] Lim H-S Choi J Lee B Kim SG Kim YS Yoo J-J. Association between inflammatory biomarkers and nutritional status in fatty liver. *Clin Nutr Res.* (2020) 9:182. 32789148 10.7762/cnr.2020.9.3.182 PMC7402975

[73] Kumar R Porwal Y Dev N Kumar P Chakravarthy S Kumawat A. Association of high-sensitivity C-reactive protein (HS-CRP) with non-alcoholic fatty liver disease (NAFLD) in Asian Indians: a cross-sectional study. *J Fam Med Prim Care.* (2020) 9:390. 10.4103/jfmpc.jfmpc_887_19 32110624 PMC7014887

[74] Annani-Akollor ME Laing EF Osei H Mensah E Owiredu E-W Afranie BO Prevalence of metabolic syndrome and the comparison of fasting plasma glucose and HbA1c as the glycemic criterion for MeS definition in non-diabetic population in Ghana. *Diabetol Metab Syndr.* (2019) 11:1–8. 10.1186/s13098-019-0423-0 30949244 PMC6431006

[75] Sprinzl MF Weinmann A Lohse N Tönissen H Koch S Schattenberg J Metabolic syndrome and its association with fatty liver disease after orthotopic liver transplantation. *Transpl Int.* (2013) 26:67–74. 23126674 10.1111/j.1432-2277.2012.01576.x

[76] Ma H Xu C Xu L Yu C Miao M Li Y. Independent association of HbA1c and nonalcoholic fatty liver disease in an elderly Chinese population. *BMC Gastroenterol.* (2013) 13:3. 10.1186/1471-230X-13-3 23294935 PMC3543719

[77] Kwak M-S Yim JY Kim D Park MJ Lim SH Yang JI Nonalcoholic fatty liver disease is associated with coronary artery calcium score in diabetes patients with higher HbA1c. *Diabetol Metab Syndr.* (2015) 7:1–6. 25844093 10.1186/s13098-015-0025-4 PMC4384364

[78] Godoy-Matos AF Silva Júnior WS Valerio CM. NAFLD as a continuum: from obesity to metabolic syndrome and diabetes. *Diabetol Metab Syndr.* (2020) 12:1–20. 32684985 10.1186/s13098-020-00570-y PMC7359287

[79] Adams LA Waters OR Knuiman MW Elliott RR Olynyk JK. NAFLD as a risk factor for the development of diabetes and the metabolic syndrome: an eleven-year follow-up study. *Off J Am Coll Gastroenterol.* (2009) 104:861–7. 10.1038/ajg.2009.67 19293782

[80] Hrncir T Hrncirova L Kverka M Hromadka R Machova V Trckova E Gut microbiota and NAFLD: pathogenetic mechanisms, microbiota signatures, and therapeutic interventions. *Microorganisms.* (2021) 9:957. 10.3390/microorganisms9050957 33946843 PMC8146698

[81] Eguchi K Kuruvilla S Ogedegbe G Gerin W Schwartz JE Pickering TG. What is the optimal interval between successive home blood pressure readings using an automated oscillometric device? *J Hyperten.* (2009) 27:1172. 10.1097/hjh.0b013e32832a6e39 19462492 PMC2941726

[82] Hashemian M Poustchi H Merat S Abnet C Malekzadeh R Etemadi A. Red meat consumption and risk of nonalcoholic fatty liver disease in a population with low red meat consumption. *Curr Dev Nutr.* (2020) 4(Suppl. 2):1413. 10.14309/ajg.0000000000001229 33767101 PMC8460710

[83] Ghaffarpour M Houshiar-Rad A Kianfar H. The manual for household measures, cooking yields factors and edible portion of foods. *Tehran Nashre Olume Keshavarzy.* (1999) 7:42–58.

[84] Fagherazzi G Vilier A Bonnet F Lajous M Balkau B Boutron-Ruault M-C Dietary acid load and risk of type 2 diabetes: the E3N-EPIC cohort study. *Diabetologia.* (2014) 57:313–20. 10.1007/s00125-013-3100-0 24232975

[85] Kiefte-de Jong JC Li Y Chen M Curhan GC Mattei J Malik VS Diet-dependent acid load and type 2 diabetes: pooled results from three prospective cohort studies. *Diabetologia.* (2017) 60:270–9. 27858141 10.1007/s00125-016-4153-7 PMC5831375

[86] Trichopoulou A Costacou T Bamia C Trichopoulos D. Adherence to a Mediterranean diet and survival in a Greek population. *N Engl J Med.* (2003) 348:2599–608. 10.1056/NEJMoa025039 12826634

[87] Remer T Manz F. Potential renal acid load of foods and its influence on urine pH. *J Am Diet Assoc.* (1995) 95:791–7. 10.1016/s0002-8223(95)00219-7 7797810

[88] Remer T Dimitriou T Manz F. Dietary potential renal acid load and renal net acid excretion in healthy, free-living children and adolescents. *Am J Clin Nutr.* (2003) 77:1255–60. 10.1093/ajcn/77.5.1255 12716680

[89] Du Bois D. A formula to estimate the approximate surface area if height and weight be known. *Nutrition.* (1989) 5:303–13. 2520314

[90] Verbraecken J Van de Heyning P De Backer W Van Gaal L. Body surface area in normal-weight, overweight, and obese adults. A comparison study. *Metabolism.* (2006) 55:515–24. 16546483 10.1016/j.metabol.2005.11.004

[91] Lipsy RJ. The national cholesterol education program adult treatment panel III guidelines. *J Manag Care Pharm.* (2003) 9(1 Suppl.):2–5. 10.18553/jmcp.2003.9.s1.2 PMC10437161 14613351

[92] Ryu E. Model fit evaluation in multilevel structural equation models. *Front Psychol.* (2014) 5:81. 10.3389/fpsyg.2014.00081 24550882 PMC3913991

[93] Beran TN Violato C. Structural equation modeling in medical research: a primer. *BMC Res Notes.* (2010) 3:267. 10.1186/1756-0500-3-267 20969789 PMC2987867

[94] Yki-Järvinen H. Non-alcoholic fatty liver disease as a cause and a consequence of metabolic syndrome. *Lancet Diabetes Endocrinol.* (2014) 2:901–10. 10.1016/S2213-8587(14)70032-4 24731669

[95] Sookoian S Pirola CJ. Shared disease mechanisms between non-alcoholic fatty liver disease and metabolic syndrome–translating knowledge from systems biology to the bedside. *Aliment Pharmacol Therap.* (2019) 49:516–27. 10.1111/apt.15163 30714632

[96] Golabi P Otgonsuren M de Avila L Sayiner M Rafiq N Younossi ZM. Components of metabolic syndrome increase the risk of mortality in nonalcoholic fatty liver disease (NAFLD). *Medicine.* (2018) 97:e0214. 29595666 10.1097/MD.0000000000010214 PMC5895395

[97] Razavi Zade M Telkabadi MH Bahmani F Salehi B Farshbaf S Asemi Z. The effects of DASH diet on weight loss and metabolic status in adults with non-alcoholic fatty liver disease: a randomized clinical trial. *Liver Int.* (2016) 36:563–71. 10.1111/liv.12990 26503843

[98] Zemel MB Teegarden D Van Loan M Schoeller DA Matkovic V Lyle RM Dairy-rich diets augment fat loss on an energy-restricted diet: a multicenter trial. *Nutrients.* (2009) 1:83–100. 10.3390/nu1010083 22253969 PMC3257590

[99] Eckel RH Jakicic JM Ard JD de Jesus JM Houston Miller N Hubbard VS 2013 AHA/ACC guideline on lifestyle management to reduce cardiovascular risk: a report of the American college of cardiology/American heart association task force on practice guidelines. *J Am Coll Cardiol.* (2014) 63(25 Pt B):2960–84. 24239922 10.1016/j.jacc.2013.11.003

[100] Akhlaghi M. Dietary approaches to stop hypertension (DASH): potential mechanisms of action against risk factors of the metabolic syndrome. *Nutr Res Rev.* (2020) 33:1–18. 10.1017/S0954422419000155 31358075

[101] Carrero JJ Gonzalez-Ortiz A Avesani CM Bakker SJ Bellizzi V Chauveau P Plant-based diets to manage the risks and complications of chronic kidney disease. *Nat Rev Nephrol.* (2020) 16:525–42. 32528189 10.1038/s41581-020-0297-2

[102] Van Name MA Savoye M Chick JM Galuppo BT Feldstein AE Pierpont B A low Ω-6 to Ω-3 PUFA ratio (N–6: N–3 PUFA) diet to treat fatty liver disease in obese youth. *J Nutr.* (2020) 150:2314–21. 10.1093/jn/nxaa183 32652034 PMC7467848

[103] Roeb E Weiskirchen R. Fructose and non-alcoholic steatohepatitis. *Front Pharmacol.* (2021) 12:634344. 10.3389/fphar.2021.634344 33628193 PMC7898239

[104] Reynolds AN Akerman AP Mann J. Dietary fibre and whole grains in diabetes management: systematic review and meta-analyses. *PLoS Med.* (2020) 17:e1003053. 10.1371/journal.pmed.1003053 32142510 PMC7059907

[105] Peng H Xie X Pan X Zheng J Zeng Y Cai X Association of meat consumption with NAFLD risk and liver-related biochemical indexes in older Chinese: a cross-sectional study. *BMC Gastroenterol.* (2021) 21:221. 10.1186/s12876-021-01688-7 34001005 PMC8127290

[106] Chauveau P Aparicio M Bellizzi V Campbell K Hong X Johansson L Mediterranean diet as the diet of choice for patients with chronic kidney disease. *Nephrol Dial Transplant.* (2018) 33:725–35. 29106612 10.1093/ndt/gfx085

[107] Abbasalizad Farhangi M Nikniaz L Nikniaz Z. Higher dietary acid load potentially increases serum triglyceride and obesity prevalence in adults: an updated systematic review and meta-analysis. *PLoS One.* (2019) 14:e0216547. 10.1371/journal.pone.0216547 31071141 PMC6508739

[108] Jayedi A Shab-Bidar S. Dietary acid load and risk of type 2 diabetes: a systematic review and dose–response meta-analysis of prospective observational studies. *Clin Nutr ESPEN.* (2018) 23:10–8. 10.1016/j.clnesp.2017.12.005 29460782

[109] Han E Kim G Hong N Lee Y-H Kim DW Shin HJ Association between dietary acid load and the risk of cardiovascular disease: nationwide surveys (KNHANES 2008–2011). *Cardiovasc Diabetol.* (2016) 15:1–14. 10.1186/s12933-016-0436-z 27565571 PMC5002186

[110] Osuna-Padilla IA Leal-Escobar G Garza-García CA Rodríguez-Castellanos FE. Dietary acid load: mechanisms and evidence of its health repercussions. *Nefrología.* (2019) 39:343–54. 10.1016/j.nefroe.2019.08.001 30737117

[111] Chan R Wong VW-S Chu WC-W Wong GL-H Li LS Leung J Higher estimated net endogenous acid production may be associated with increased prevalence of nonalcoholic fatty liver disease in Chinese adults in Hong Kong. *PLoS One.* (2015) 10:e0122406. 10.1371/journal.pone.0122406 25905490 PMC4407987

[112] Nguyen GC Thuluvath PJ. Racial disparity in liver disease: biological, cultural, or socioeconomic factors. *Hepatology.* (2008) 47:1058–66. 18302296 10.1002/hep.22223

[113] Liu S-H Lazo M Koteish A Kao W Shih M-H Bonekamp S Oral contraceptive pill use is associated with reduced odds of nonalcoholic fatty liver disease in menstruating women: results from NHANES III. *J Gastroenterol.* (2013) 48:1151–9. 10.1007/s00535-012-0715-8 23188092 PMC4170913

[114] Hajhashemy Z Shahdadian F Moslemi E Mirenayat FS Saneei P. Serum vitamin D levels in relation to metabolic syndrome: a systematic review and dose–response meta-analysis of epidemiologic studies. *Obes Rev.* (2021) 22:e13223. 33829636 10.1111/obr.13223

